# Cardiac Cx43, Cx40 and Cx45 co-assembling: involvement of connexins epitopes in formation of hemichannels and Gap junction channels

**DOI:** 10.1186/s12860-016-0118-4

**Published:** 2017-01-17

**Authors:** Thomas Desplantez

**Affiliations:** 1 IHU Liryc, Electrophysiology and Heart Modeling Institute, Fondation Bordeaux Université, Campus X. Arnozan, Avenue Haut Leveque, 33600 Pessac- Bordeaux, France; 2 Univ. Bordeaux, Centre de recherche Cardio-Thoracique de Bordeaux, U1045, F-33000 Bordeaux, France; 3 INSERM, Centre de recherche Cardio-Thoracique de Bordeaux, U1045, F-33000 Bordeaux, France

## Abstract

**Background:**

This review comes after the International Gap Junction Conference (IGJC 2015) and describes the current knowledge on the function of the specific motifs of connexins in the regulation of the formation of gap junction channels. Moreover the review is complemented by a summarized description of the distinct contribution of gap junction channels in the electrical coupling.

**Results:**

Complementary biochemical and functional characterization on cell models and primary cells have improved our understanding on the oligomerization of connexins and the formation and the electrical properties of gap junction channels. Studies mostly focused cardiac connexins Cx43 and Cx40 expressed in myocytes, while Cx45 and Cx30.2 have been less investigated, for which main findings are reviewed to highlight their critical contribution in the formation of gap junction channels for ensuring the orchestrated electrical impulse propagation and coordination of atrial and ventricular contraction and heart function, whereas connexin dysfunction and remodeling are pro-arrhythmic factors. Common and specific motifs of residues identified in different domain of each type of connexin determine the connexin homo- and hetero-oligomerization and the channels formation, which leads to specific electrical properties.

**Conclusions:**

These motifs and the resulting formation of gap junction channels are keys to ensure the tissue homeostasis and function in each connexin expression pattern in various tissues of multicellular organisms. Altogether, the findings to date have significantly improved our understanding on the function of the different connexin expression patterns in healthy and diseased tissues, and promise further investigations on the contribution in the different types of connexin.

## Background

Gap junction channels (GJCs) mediate direct intercellular coupling (electrical, metabolic) that regulates tissue homeostasis and function. They are formed by the docking of adjacent hexameric hemichannels (connexons) made of connexins (Cxs) inserted in the membrane of neighboring cells. Currently, 21 isoforms of Cxs have been identified in the human genome and 20 in the mouse genome [1–3]. These are largely orthologous genes classified as α, β and “other” (γ,δ,ε) gene families [3] according to the degree of amino acid sequence homology. Cxs have a similar membrane structural topology [4] with four transmembrane domains (M1-M4), two extracellular loops (E1, E2), one amino cytoplasmic domain (NTH), one cytoplasmic loop (CL) and one carboxy (CH) cytoplasmic domains. A high degree of homology has been observed between E1, E2 and the interface region of CL and M3. On the contrary, COOH, NTH and CL domains have a lower degree of homology. These different degrees of homology are attributed to different functions in the regulation and the biophysical properties of GJCs.

The regulation of the formation of GJCs has been elucidated by the use of complementary biochemical techniques (e.g. immunofluorescence, western blot) on various types of wild-type and altered Cxs (mutated, truncated, chimeric, etc.) and biological tools (transfected cell models or native primary cells). Biochemical techniques have revealed the junctional co-localization of Cxs that suggested the formation of GJCs made of diverse types and amount of Cxs. However, limitations in frame resolution and observation level (i.e. multichannel *vs* single channel) precludes further characterization of the Cxs composition of GJCs. However, the functional approaches, more especially by the use of the dual voltage clamp recordings on cell pairs, permitted to overcome this limit and further demonstrates the regulated formation of GJCs and described the formation of homotypic, heterotypic and heteromeric GJCs, and illustrated whether GJCs are functional in the different Cx expression patterns. In addition, single channel recordings enable a finer determination of the type of GJC formed and estimate their Cxs composition.

A significant amount of literature is available on the regulated formation of GJCs, highlighting the conjugated function of common and specific motifs located in different domains of Cxs. A main part of these findings are reviewed in [5–7], which are important to correlate the function of the different Cx expression patterns to tissue homeostasis and function and to understand why Cxs dysfunction and remodeling are pro- pathological.

This review summarizes the current knowledge on the epitopes implicated in the regulation of cardiac GJC formation, covering the published literature (referenced throughout this manuscript) and new findings reported during the International Gap Junction Conference in 2015 (IGJC 2015). More precisely the review will focus on the formation of cardiac GJCs made of Cx43, Cx40 and Cx45. Briefly, it is well known today that these 3 Cxs have specific expression patterns that, importantly, display varying levels of co-expressions that leads to different ratios of Cx43:Cx40, Cx40:Cx45 and Cx43:Cx45 in the healthy heart since the embryonic stage, which are altered in the diseased heart, termed Cxs remodeling [8]. Until now, most studies focused on Cx43 complemented by recent findings on Cx40 while Cx45 has been less explored. These studies have identified in each Cxs isoform different and common motifs implicated in the regulation of the homo or hetero-assembling during the intracellular trafficking. Moreover, the Cxs assembling appears dependent on the relative level of expression of each isoform. This makes further study on Cx45 (mostly expressed at significant lower levels than Cx43 and Cx40) critical, demonstrated by the electrical properties of GJCs observed in cell lines and cardiac myocytes co-expressing Cx45 with Cx43 and/or Cx40 that do not match this assumption. This reflects an additional regulation for ensuring the specific formation and functional contribution of certain types of GJCs critical for the cardiac function.

### Cardiac Cxs co-expression and diversity of gap junction channels

Before describing the function of different motifs and residues regulating GJCs formation, this section describes the cardiac Cx expression patterns that are highly complex with four types of Cxs expressed and varying distributions and levels of expressions, which are critical for ensuring cardiac function.

### Normal connexin expression pattern in cardiac tissue

**Fig. 1:**
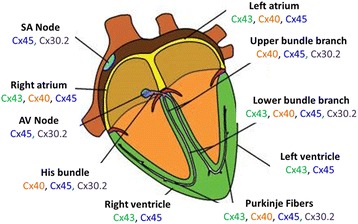
cardiac Cxs patterns of expression. Note the ubiquitous co-expression of 2, 3 and 4 Cxs isoforms. (The abbreviated anatomical regions are defined at the beginning of the manuscript). (modified from Severs et al, 2008)

Four cardiac Cxs, Cx40, Cx43, Cx45 and Cx30.2, exhibit a specific spatio-temporal expression pattern (Fig. 1). An important observation has been the co-expression of Cxs in 2–4 isoforms in each specialized tissue (e.g. ventricular myocytes, atrial myocytes and the conduction system) that display changing levels of expression (e.g. embryonic stage, post-natal). Cx45 is the first Cx expressed during development with the apparition of the first cardiac contractions and exhibits a ubiquitous expression in the developed cardiac tissue [8–10]. Other Cxs are expressed later during development in a more specific pattern [8, 9]. For example, Cx30.2 is specific to the conduction system [11] while Cx43 is mainly expressed in atrial and ventricular myocytes and less expressed in the conduction system such as Purkinje Fibers [8]. The progressive and chronological increase of the expression of Cx43 and Cx40 is conjugated to decrease of the level of expression of Cx45 that is then expressed at significantly lower levels than other Cxs [12, 13] making this Cx difficult to characterize in terms of accurate cell distribution and levels of expression due to the lower threshold of detection of the biochemical approaches such as immunofluorescence [14]. Interestingly it seems that the expression pattern varies with species: for instance the orthologous of mCx30.2 in human hCX31.9 is not observed in the conduction system [15].

Moreover, biochemical studies have shown that Cxs exhibit a graduated distribution. For example Cx43 and Cx45 are distributed in two connecting compartments in the rabbit atrioventricular conduction axis [16], and in ventricular myocytes Cx45 appears mostly localized in the endocardial area while Cx43 appears distributed in the endo-, mid- and epi-cardium [8].

### Diseased connexin remodeling

The dysfunction of cardiac Cxs has been found to be a main pro-arrhythmic factor. It is characterized by an alteration in Cx distribution and level of expression, termed Cxs remodeling, which is increasingly observed in patients with various cardiac diseases such as heart failure and atrial fibrillation (AF) [17–21]. However, much remains to be understood about the relationship between remodeling and the triggering and maintenance of these diseases. A stricking case is the research into AF that is relatively advanced but still largely misunderstood as illustrated by various remodelings of Cx40 and Cx43 observed by immunofluorescence and western blotting in patients and animal models with the same type of AF [22–25]. However, Cx45 is still less explored. To explore the specific contribution of Cx45 the use of knock-out Cx45 animal model is not possible because KOCx45 die shortly after birth [26]. This prevents the cell isolation for biochemical and functional characterization of GJCs at different age stages. However, the development of the transgenic mouse with the Cre-lox system that permits to induce Cx45 deletion [27] is promising for such further characterization [28] by performing electrical recordings on pairs of isolated myocytes that will be correlated to the biochemical characterization of Cxs expression patterns.

A possible reason of this misunderstanding is that most of the remodelings were observed from biochemical approaches using tissues samples, whereas fewer functional studies have been performed on tissues, primary cardiac myocytes and cell models.

### Ratio and diversity of gap junction channels

**Fig. 2:**
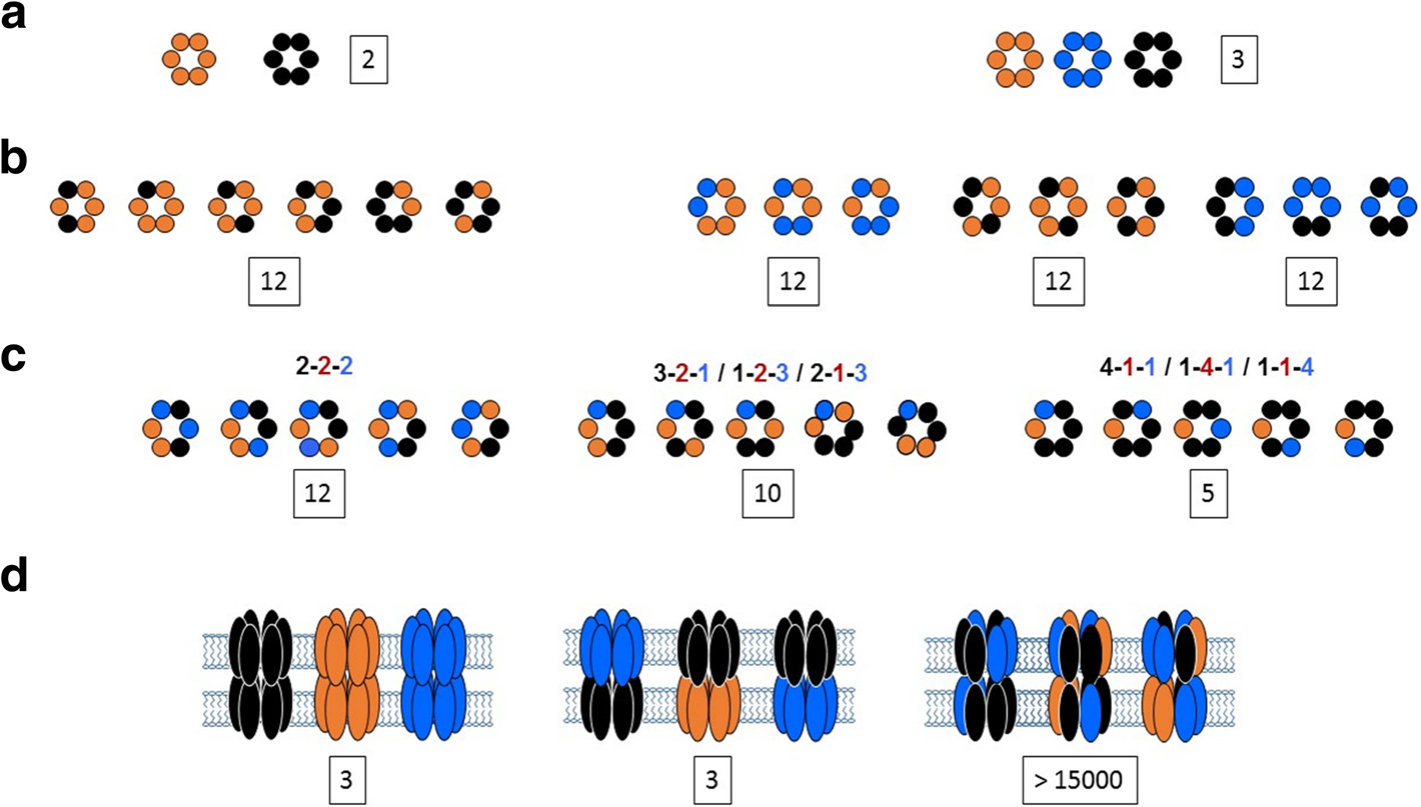
connexin composition of connexons and GJCs in cells co-expressing 2 and 3 Cxs isoforms. **a**: homomeric connexons: double Cxs co-expression (left panel) and triple Cxs co-expression (right panel). **b**: examples of diverse Cxs compositions of heteromeric connexons made of 2 isoforms: double Cxs co- expression (left panel) and triple Cxs co-expression (right panel). **c**: examples of diverse Cxs compositions of heteromeric connexons made of 3 isoforms. The colored numbers indicate the relative amount of Cxs isoforms. **d**: diverse types of GJCs for a triple Cxs co-expression: homotypic (left panel), heterotypic (middle panel), and heteromeric (right panel). The numbers in frames indicate the statistical total amount of each configuration

Both healthy and diseased expression patterns induce the presence of a heterogeneous distribution of Cxs in each tissue, leading to different varying ratios of co-expressed Cxs where different types of connexons and GJCs can be statically formed (Fig. 2) [29, 30]. The nomenclature of GJC has been demonstrated to be a function of their composition. Briefly, homotypic GJCs refer to a single and same Cx composition in both docked connexons, while heterotypic and heteromeric GJCs are composed of mixed Cx composition. Most of the time heterotypic GJCs refer to channels made of homomeric connexons composed of a single different type of Cx in each connexon, and heteromeric channels to GJCs made of connexons with mixed Cx composition in one or both connexons. In total, 196 GJCs and more than 15000 are statistically possible in cells co-expressing 2 and 3 Cxs, respectively (Fig. 2). This complex diversity and broad statistical possibility of Cxs (co-)assembling makes it critical to better understand how specific types of GJCs are formed instead of a stochastic formation in each differentiated tissue to ensure the intercellular communication in the healthy heart, and understand how Cx remodeling leads to cardiac diseases such as AF. An estimation of the statistical amounts of all differnet possible Cxs composition of hemichannels and gap junction channels are summarized in Tables 1 and 2 for respectively 2 and 3 co-expressed Cxs.

**Table 1:** Statistical amounts of hemichannels and gap junction channels in the case of 2 co-expressed connexins

	Double co-expression of Connexins	
	Hemichannels	Gap junction channels
	2 homomeric 12 heteromeric	2 homomeric-homotypic 1 homomeric-heterotypic 24 heteromeric-homotypic 168 heteromeric-heterotypic
Total	14	195

These amounts consider that each association is possible independently on the connexin composition of hemichannels, and are indicated without considering that each channel can be in 2 different orientations

**Table 2:** Statistical amounts of hemichannels and gap junction channels in the case of 3 co-expressed connexins

Hemichannels	Gap junction channels
Homo-oligomerization	Homomeric-homomeric docking: homotypic channels
3	3
Hetero-oligomerization	Homomeric-homomeric docking: heterotypic channels
2 isoforms: 36 (see double co-expression) 3 isoforms: 2:2:2 ratio: 12 3:2:1 ratio: 30 4:1:1 ratio: 15	3
	Homomeric-heteromeric docking: heterotypic channels
	3 x 93 = 279
	Heteromeric-heteromeric docking: homotypic channels
	93 (one per composition)
	Heteromeric-heteromeric docking: heterotypic channels
	36x12x30x15 – (279 + 93) = 194 028

These amounts consider that each association is possible, independently on the connexins composition of hemichannels made of 2 and 3 connexin, and are indicated without considering that each channel can be in 2 different orientations

## Results

### Cardiac Cxs: epitopes and formation of connexons

**Fig. 3:**
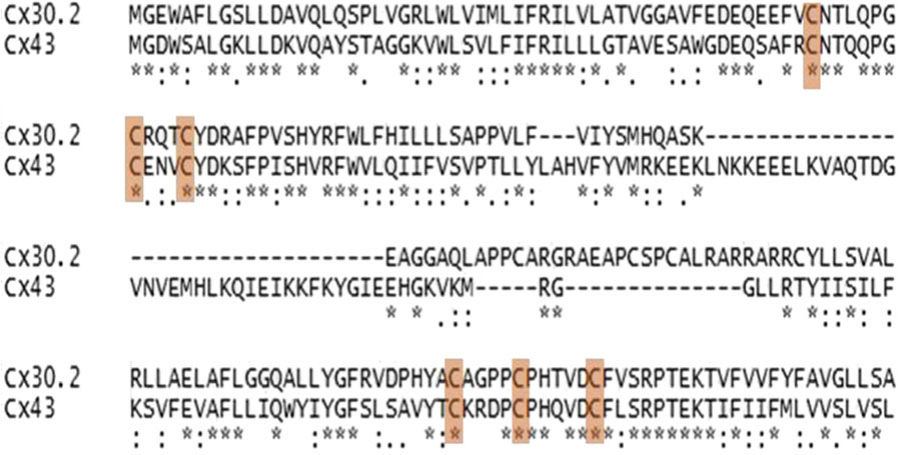
Triplets of Cys identified in the EL1 and EL2 domains of mCx30.2. Note that illustrates the high conservation of this motif between the 4 cardiac Cxs. The sequence alignment with Cx43 has been made with MAFFT software

Different Cxs constructs (e.g. chimeric, truncated, tagged) have been made and transfected in cell models to elucidate the function of domains and motifs in the formation of connexons and GJCs. In cardiac tissue, most data was gained from studies on Cx43 and Cx40 whereas Cx45 and Cx30.2 have been less explored the principal motifs and residues identified in the Cxs oligomerization and stability, and hte foramtion of gap junction channels are indicated in Tables 3 and 4. Connexon formation occurs in the Endosplasmic Reticulum (ER) and the Trans-Golgi Network (TGN) (see below) and is regulated by criteria reviewed in the following paragraphs. As presented in Section 3 - Ratio and diversity of gap junction channels and Fig. 3, co-expression leads to a statistical dominant hetero-oligomerization, also observed in-vitro and *ex-vivo*.

**Table 3:** Residues and domains implicated in the stability and the oligomerization of connexins for cardiac Cx40, Cx43 and Cx45 expressed in myocytes

	Connexins		Oligomerization of connexins			
	Stability of connexins		Assembly signal		Selectivity signal	
	Domain	Motif	Domain	Motif	Domain	Motif
Cx40	E2 *M3* *(cytoplasmic end)*	Assumed similar to Cx43 LL**N**TY	*M3*	L**N** [38]	n.d. : assumed similar to Cx43	
Cx43	E2 M3 (cytoplasmic end)	YGF [5, 31, 36] LL**R**TY [37, 38]	*M3*	151-L**R**-154 [37]	NTH (position 11,12) M3	DK [37, 39, 45] L**R**
Cx45	E2 M3 (cytoplasmic end)	Assumed similar to Cx43 LL**K**TY	n.d. : assumed similar to Cx43: L**K**		n.d. : assumed similar to Cx43	

In bold and underlined are specific residues implicated in the fine regulation (see text for more details). For Cx40 and Cx45 a similar function of motifs not identified yet is assumed (e.g. YGF in E2 of Cx43 implicated in the stability of connexins). n.d.: not determined

**Table 4:** Residues and domains implicated in the stability and the docking of hemichannels for cardiac Cx40, Cx43 and Cx45 expressed in myocytes

	Stability and docking of hemichannels	
	Domains	Motifs
Cx40	NTH E1 E2	Similar to the motifs ensuring the stability of connexins ExxxE [62–64] **D**xQI 53-CxxxxxxCxxC-66 (mCx40) VxxxxxxxH**P**xN 184-CxxxxxxCxxxC-197 (mCx40)
Cx43	NTH E1 E2	Similar motifs to the motifs ensuring the stability of connexins KxxxK [62–64] **N**xLQ 53-CxxxxxxCxxC-65 (mCx43) TxxxxxxxHQxD 186-CxxxxxxCxxxC-199 (mCx43)
Cx45	NTH E1 E2	Similar motifs to the motifs ensuring the stability of connexins RxxxE [65] **N**xLQ 52-CxxxxxxCxxC-63 (mCx45) VxxxxxxxH**K**xD 208-CxxxxxxCxxxC-221 (mCx45)

In bold and underlined are specific residues implicated in the fine regulation of the formation of GJCs (see text for more details). n.d.: not determined

### Stability of connexins and oligomerization

#### Stability of connexins

A first determinant criterion required for connexon formation is the stability of Cxs as monomers during intracellular trafficking. Studies have mostly focused on Cx43 and have shown that oligomerization occurs in the TGN [5, 31] similarly to other isoforms such as Cx26 and Cx46 [32–34] while other isoforms such as Cx32 oligomerize in the ER. These different localizations are mainly explained by the different intracellular pathways that follow Cxs, but also depend on the functions ensured by the motifs [5, 35].

Two main motifs have been identified for Cx43: i) YGF in the E2 domain that confers a specific structure to a binding site for interaction with the chaperone protein ERp29 [5, 31, 36], and ii) LLRTY near the cytoplasmic end of the M3 domain, also present in most α Cxs, especially the central R that confers a helical conformation to the domain [37, 38]. Similarly, a conserved K in the δ Cxs is used to classify Cxs as the R type that comprise Cx43 and Cx45, and a conserved doublet WW in the β Cxs, classifying the W type [7, 39]. More precisely, in Cx45 K is sequenced instead of R, both having similar physical properties (amino group and charge) that are believed to confer to the motif an identical configuration (e.g. stretch between residues) in Cx43 and Cx45 and ensuring their high efficiency of hetero-oligomerization. Similarly the sequence LLNTY has been identified in Cx40 classified in a third group named “other”. In this sequence the uncharged N in Cx40 might induce a different configuration to the motif and consequently a lower Cx43/Cx40 hetero-oligomerization compatibility, as demonstrated by a lower electrical coupling than their homotypic counterparts [40]. A similar assumption can be advanced for the Cx40/Cx45 hetero-oligomerization compatibility.

The concomitant function of these motifs stabilizes the positioning of the M3 domain that prevents early oligomerization of Cx43 in the ER and permits its traffic until the TGN where it hetero-oligomerizes with other Cxs. Importantly, the flexible regulation of the hetero-oligomerization between the R and the Other groups suggests that Cx45 follows the same intracellular pathway as Cx43 and Cx40 that assures the specific formation of GJCs. What needs to be determined is the regulatory mechanism of the separation of Cx43 and ERp29 prior to oligomerization. A principal hypothesis is that the change of environment (e.g. pH) in the cytoplasmic compartments regulates the stability of the link between ERp29 and Cx43. Moreover, what is still unknown is if the trafficking of Cx45, Cx40, and Cx30.2 depends on an interaction with a chaperone protein (similar or different to ERp29) or another regulatory mechanism.

#### Connexons and oligomerization: assembly signal

Studies on truncated and mutated Cx43 have revealed that the motif in the M3 domain (COOH terminal portion that stabilizes Cxs as monomer in the ER) indirectly regulates the hetero-oligomerization in the TGN by ensuring the correct positioning of the M3 domain. Both R- and W-type Cxs have non-conserved residues that represent the *assembly signal* [37, 39]. For instance the R type Cx43 has a doublet LR at positions 152 and 153 [37] that permits Cxs to oligomerize each other and the formation of homomeric connexons. Such regulation likely also occurs for hetero-oligomerization, which suggests a minimum of two identical Cx isoforms in connexons and reducing the number of connexons and GJCs formed in cells co-expressing Cxs. Similarly, this motif most likely regulates the homo-oligomerization of “other” type of Cxs, such as the hetero-oligomerization of Cx40 that contains a doublet LN [38] (see paragraph 4.c). By extension we can also assume a similar residue sequence and function in Cx45 and Cx30.2, as suggested by the diverse functional cardiac GJCs observed [11, 30, 40–44].

#### Connexons and oligomerization: selectivity signal and heteromeric compatibility

The formation of heteromeric connexons represents a higher level of complexity in the Cx machinery as this requires recognition and compatibility between the different isoforms regulated by motifs identified in the Cx gene groups. Importantly, hetero-oligomerization depends on the gene group to which Cxs belong to, and for instance no hetero-oligomerization has been observed between α and β Cxs.

A *selectivity signal* firstly identified in Cx43 by different approaches such as directed mutagenesis, truncation of Cxs and sequences alignment [37, 39, 45] is composed of conserved amino acid located in the NTH and M1 domains: i) doublet DK at positions 11 and 12 of the NTH domain [37], and ii) the central R in the LLRTY sequence also implicated in the stability of Cxs as a monomer [37, 38] and the homo-oligomerization. The multiple functions ensured by this motif have suggested an indirect regulation [38, 46] based on the hypothesis that it permits the formation of salt bridges and hydrogen bonds (*see B.2. Stability of connexons*). Interestingly, this restriction between alpha and beta groups can be disrupted in case of mutation in the NTH domain. This has been recently observed between Cx26 and Cx43 [47] in case of mutation in the NTH domain of Cx26 that causes keratitis-ichthyosis-deafness. Indeed this mutation leads to altered and aberrant properties of heteromeric connexons made of Cx26/Cx43 and nonfunctional GJCs. This reinforces the critical function of this domain in regulating the Cxs co-assembling and the formation of connexons.

The low degree of identity of the residue sequences observed between the α and β Cxs prevents their hetero-oligomerization, whereas the hetero-oligomerization between the α and the “other” group occurs, suggesting a similar folded motif (residues, structure, charge, etc.). This is determinant for cardiac function, more particularly for Cx45, classified as “other” Cxs and that, despite its low level of expression, oligomerizes with the atrial Cx43, Cx40 and ventricular Cx43 in myocytes. Indeed in both cell types the GJCs that govern the electrical coupling have been characterized as heteromeric (see further; [48, 49]).

To further investigate this regulation we recently developed the model of Rat Liver Epithelial cells (RLE) stable transfected with an inducible Ecdysone system that permits to control the level of co-expressed Cxs and their ratios. Two cell lines have been created co-expressing Cx43 + Cx40 and Cx43 + Cx45 named Ind40 and Ind45. Interestingly in both cell lines we observed a low degree of hetero-oligomerization relative to the total Cx content whereas these heteromeric channels dominate the electrical coupling ([50, 51]; see further).

When correlated to the formation of GJCs in the different specialized tissues and the changing Cx levels of expressions in the healthy and diseased heart, it is important to take into account that the oligomerization of Cx43 and Cx40 is saturable [52], i.e. when overexpressed, early oligomerization of the over-expressed isoform can occur in the ER. This illustrates a dependence on levels of expression and a specific contribution of Cx43 and Cx40 to ensure specific formation of GJCs in the healthy heart while pathological Cxs remodeling, as observed for example in AF [25], might alter this machinery and function. Importantly, the low level of expression of Cx45 that could suggest a negligible function and contribution appears critical in this regulation as the heteromeric GJCs made of Cx40 and Cx43 seem to form only when Cx45 is present [43]. In the same way the dominant contribution of heteromeric Cx43/Cx45 in ventricular myocytes agrees the critical function of Cx45 despite a low level of expression [12, 48, 53].

### Stability of connexons

Similarly to stability of monomeric Cx required during intracellular trafficking, the *stability of connexons* is essential to ensure their insertion in the cell membrane and the translocation toward the perinexus and gap junction plaque [54]. Further, as previously described, the motif of R, W and other types of Cxs, indirectly controls the stability of connexons by forming non-covalent bounds (e.g. salt bridges and hydrogen bonds) [38, 46] in the M2 and M4 domains that confer precise conformation of the domains.

In addition, a primary sequence analysis (e.g. [55, 56]) and mutation approach have identified conserved triplets of Cysteine C-C-C in E1 and E2 domains of Cx43, Cx40 and Cx45 that form disulfide bonds between E1 and E2 of a single Cx respectively [57] that stabilize the connexons. Importantly, these residues are highly conserved between connexins isoforms and their position (see table for position in mCx40, mCx43 and mCx45) is critical to ensure the docking of hemichannels. Because these bonds are formed between C residues of the same Cx monomer, we can assume that this regulation occurs for both the homo- and heteromeric connexons. In complement, as suggested by the observation of functional homotypic, heterotypic and heteromeric GJCs made of Cx30.2 [11, 44, 58] the same motif is identified in Cx30.2 (Fig. 3).

### Formation of gap junction channels

Homotypic, heterotypic and heteromeric GJCs are formed from the docking of adjacent connexons inserted in the plasma membrane of neighboring cells. Complementary approaches such as high resolution structure, point mutations and sequence alignment have concluded that the docking is mainly regulated by motifs in the E1 and E2 domains that have common and specific functions. Most of studies have focused on GJCs of mixed Cxs composition, i.e. heterotypic and heteromeric GJCs, and a similar regulation is assumed for homotypic channels.

### Hydrogen bonds

#### Residue triplets

*Residue triplets* at positions 54, 56 and 57 in E1 form non-covalent E1-E1 hydrogen H-bonds between two Cxs monomers that face each other in adjacent connexons [6, 7]. The NxLQ sequence has been identified in human hCX43 and hCX45. A sequence alignment with cardiac hCX40 and mCx30.2 identified the same sequence as hCX43 in mCx30.2 and the DxQI sequence in hCX40 (Bai & Wang, 2014). Importantly, a minimum number of H-bonds, up to an estimated maximum of 24, is necessary to ensure the positioning of adjacent connexons that adopt a rotated 30° angle and “peak and valley” arrangement [59] and the E1 domain to line the inner surface of the channel and the channel pore. This regulatory factor of the hetero- compatibility suggests different efficiencies of functional GJCs in function of their Cx composition.

#### Residues 167, 176, 177 and 179

The human hCX26 has been the first human Cxs to be investigated. Studied have identified a the sequence KxxxxxxxNTxD at positions 167, 176, 177 and 179 in the E2 domain that has a similar function as the motif in E1. A sequence alignment with human cardiac Cxs identified i) VxxxxxxxHPxN in hCX40, ii) TxxxxxxxHQxD in hCX43, and iii) VxxxxxxxHKxD in hCX45 [6], and AxxxxxxxHTxD in hCX31.9 (when aligned with hCX43). Point mutations and structure model studies have shown, similarly to the motif in E1, that a minimum number of H-bonds is necessary with an estimated maximum of 36. In addition, as previously demonstrated, the E2 domain is highly implicated in regulating the heterotypic compatibility of Cxs.

Altogether, the E1-E1 and E2-E2 H-bonds concomitantly formed between adjacent Cxs stabilize the connexons and ensure their docking and the channel function. As already proposed for the other motifs, the properties of the amino group of residues confer a precise configuration (e.g. stretch, arrangement, and total charge) to the domains necessary for the formation of the H-bonds that most likely contribute indirectly to making Cxs compatible or incompatible, for example by a charge repulsion or a charge attraction, which is complemented by the specific function of other motifs for the heterotypic and heteromeric compatibility (see below).

### Heterotypic compatibility

The formation of GJCs has been mainly elucidated by the observation of their functionality, mostly by electrical recordings on cell pairs with native and mutated Cxs to correlate the function of motifs to the function of GJCs. The motifs implicated in the formation of H-bonds in the E2 domain (see previous paragraph) also regulate *heterotypic compatibility* and are used to classify Cxs. The four cardiac Cxs are classified as H type and form functional heterotypic channels all together that provide different efficiencies of electrical coupling [30, 41, 43]. In addition a K-N and an Other class have been established [60]; reviewed in [6, 7]).

The different electrical and metabolic properties of heterotypic cardiac GJCs are function of the types of Cxs forming the channels, among them the amplitude of the electrical coupling differs to homotypic GJCs (also observed for heteromeric GJCs – see further). This indicates a regulation of the degree of heterotypic compatibility and amount of coupling mediated by GJCs as a function of the Cx expression pattern to ensure the different conduction velocities of the electrical impulse and the coordinated atrial and ventricular contractions. In this sense the functional cardiac heterotypic GJCs, even for the controversial Cx43/Cx40 configuration, indicate a compatibility unrelated to gene family and a flexibility between the four cardiac Cxs, on the contrary to the heteromeric compatibility (see below). Interestingly, the least compatibility was observed with Cx40 [42, 43] that suggests a limited formation regulated by Cx40 that is most likely related to higher differences in residue sequences in the motifs with other Cx isoforms. In this sense, the recent data of Jassim and colleagues [61] confirmed the critical function of the residue by observing a higher heterotypic Cx43/Cx40 compatibility when exchanging D55 and P193 of Cx40 with N and Q of Cx43. This is determinant for cardiac function, especially in atrial myocytes that co-express large amounts of Cx43 and Cx40 and traces of Cx45, making the question of the dependence on the level of expression advanced by previous studies to be extrapolated to the type of Cxs, the cell type and function. By extension we can assume that N in E1 domain and K in E2 domain of Cx45 play the same function.

Furthermore, the heterotypic compatibility occurs also between single and co-expressing Cx43 and Cx40 cells [41] and between single expressing Cx43 and Cx45 cells with co-expressing Cx43 + Cx45 cells [30]. This is determinant for cardiac function at the frontier between regions of different expression patterns such as the SAN/atrial myocyte junction [8] where similar patterns are present. Further investigations along these lines, more particularly on Cx45, the less “biochemically” studied cardiac Cx, are still necessary to better determine its function in regulating the the co-assembling compatibility and the formation of different GJCs and (see further).

### Heteromeric compatibility

The formation of heteromeric GJCs increases the complexity of the Cx machinery as it depends on the specific affinity and compatibility between heteromeric connexons. The incompatible hetero-oligomerization between α, β and the Other Cxs group might prevent abnormal metabolic and electrical coupling in a same tissue and between different bordered tissues that could alter the tissue function and homeostasis.

While most studies have identified the regulatory motifs for the formation of heteromeric connexons, fewer studies have elucidated whether additional motifs to the ones that ensure a “common” function in the formation of GJCs regulate the docking of heteromeric connexons. It is tempting to assume that the same motifs as the ones implicated in the formation of heterotypic GJCs regulate the compatibility of heteromeric connexons. In complement to the structural function of the motifs in the NHT domain that line the pore entrance of channels and form a “funnel”, a motif firstly for cardiac Cx43 and Cx40 identified in different species such as human, chicken and rodent, regulates the functionality and properties - such as the unitary conductance - of heteromeric GJCs [6, 7, 62–64] at positions 9–13 identified by KxxxK in Cx43, ExxxE in Cx40, RxxxE in mCx45 [65] and SxxxA in mCx30.2. Interestingly, the interactions between the NTH domains of different Cxs are believed to limit the functionality of heteromeric Cx40/Cx43 but not their formation [40, 65].

To further explore this point we have developed the model of homomeric and heteromeric tandem cardiac Cxs. They consist in 2 Cxs (Cx1-Cx2) linked with their entire coding sequences (except the STOP codon of Cx2) to force the expression of dimers of Cxs and reduce the number of homomeric and heteromeric connexons and channels formed [42, 66, 67]. Our electrical recordings by dual voltage clamp confirmed the contribution of hetero-domain interaction illustrated by various electrical properties (voltage gating) in function of the type of Cxs and how they assemble in tandems (Desplantez et al: presented at IGJC 2005 and 2007 : [66, 67]).

## Discussion and conclusions

### Functional contribution of cardiac gap junction channels

The regulated formation of GJCs by the specific motifs in each connexin is a critical key for regulating their function that is structural (e.g. organization and stability of the gap junction plaque) and functional (metabolic and electrical coupling). Indeed this ensures the formation of various types of GJCs in each tissue related to the expression pattern that conducts to specific determinant properties to regulate the tissue function and homeostasis.

In this sense one major role of cardiac GJCs being to regulate the electrical cell-cell coupling and impulse propagation, it appears important to summarize the current knowledge on the different contribution of GJCs related to their formation.

### Homotypic, heterotypic and heteromeric gap junction channels

To improve our understanding on this function the RLE Ind40 and Ind45 cell lines have been developed and used for electrophysiological recordings on cell pairs [50, 51, 68]). These models permitted to quantify the homo- and hetero-Cxs assembling and relate to the contribution to the electrical coupling [50, 51]. The types of channels have been identified by single channel recordings with as reference the known conductances of homotypic Cx43, Cx40 and Cx45 channels [41, 69–71]. The data obtained were then compared to electrical recordings performed on freshly isolated atrial and ventricular myocytes, taking in account that the triple Cx43/Cx40/Cx45 co-expression in atrial myocytes might induce a different formation of channels to the one characterized in the Ind40 (Cx43 + Cx40) cell line. Similarly, ventricular myocytes and Ind45 cell line express similar Cxs (Cx43 + Cx45) but different ratios.

#### Homotypic Gap junction channels

In atrial myocytes we observed that homotypic GJCs contribute at least at 25% to the electrical coupling and are mostly represented by homotypic Cx40 and Cx43 whereas homotypic Cx45 GJCs were less identified (<5%) [49]. Interestingly this differed from the amount quantified in Ind40 cells where we observed a dependence on the ratio of Cx43:Cx40: a dominant contribution of homotypic Cx43 channels when Cx43 is more expressed than Cx40 (junctional ratio Cx43:Cx40 ≈ 2), whereas higher expression of Cx40 (junctional ratio of Cx43:Cx40 ≈ 1 [50, 51]) decreased this contribution in favor of the contribution of heteromeric Cx43/Cx40 channels (see below). Interestingly, independently on the ratio of Cx43:Cx40, a similar junctional content of Cx43 + Cx40 was observed, which suggested a fine regulation of Cxs co-assembling not directly linked to their junctional content. As noticed the different Cxs expression patterns between atrial myocytes and Ind40 cells suggests an additional regulation of formation and contribution of GJCs in function of the amount of co-expressed Cxs in cells, which reveals in this case a determinant role of Cx45 despite a low levels of expression.

In ventricular myocytes homotypic channels contribute poorly (< 5%) to the electrical coupling [48], similar to our observation on Ind45 cells where our data suggest that this minor contribution does not depend on the level of expression of Cx43 and Cx45 and the ratio of Cx43:Cx45. This confirms the determinant role of Cx45 in heteromeric compatibility despite low levels of expression that warrants further investigations.

#### Heterotypic Gap junction channels

Similarly to homotypic GJCs a poor contribution of heterotypic GJCs (<5%) has been noticed between myocytes. It is tempting to believe that a higher contribution might occur at the border zones between cell types (e.g. fibroblast/myocytes) and tissues (e.g. nodal cells/atrial myocytes) expressing different sets of Cxs that favors their formation.

This was particularly suggested by electrical recordings between wild-type (Cx43^+/+^) and knock-out Cx43 (Cx43^-/-^) ventricular myocytes where similar properties to heterotypic Cx43/Cx45 GJCs not observed in pairs of Cx43^+/+^ and Cx43^-/-^ myocytes [30, 42, 53] have been observed. However, in atrial myocytes none properties of heterotypic channels of any Cxs composition have been observed, which does not exclude their contribution but, in this case, would be relatively poor. This opens the question of how and in which condition Cxs co-assemble or not during trafficking (e.g. Cxs expression pattern), is the docking between hemichannels function of their Cxs composition and affinity, and which factors (e.g. residues motifs) regulate the functional contribution of specific types of GJCs? In this sense the changes of levels of Cxs expression in the healthy and diseased heart have to be carefully further considered as a regulating factor.

#### Heteromeric Gap junction channels

A dominant contribution of heteromeric GJCs in atrial and ventricular myocytes (Beauchamp et al., 2004; Desplantez et al., 2012) was observed, which does not depend on the pattern of Cxs as we observed a similar contribution in wild-type Cx43^+/+,^ heterozygotes Cx43^+/-^ and knock-out Cx43^-/-^ genotypes [49]. This is similar to our quantified functional contribution in Ind45 cell line independently on the level of expression of Cx43 and Cx45 and their ratio, but differed to the contribution in the Ind40 cell line where the higher the level of expression of Cx40, the higher the contribution of heteromeric channels. But again the absence of Cx45 might influence this regulation and contribution. Importantly the biochemical characterization of GJCs in Ind40 and Ind45 cell lines indicated a poor Cxs co-assembling [51]. Assuming a similar amount in myocytes would indicate a fine regulation between the types and the contribution of channels. Interestingly, this dominant function leads to a nearly symmetrical voltage gating of GJCs that suggests a specific arrangement of GJCs of different or similar Cxs composition and a regulation of the docking of connexons. This raises the question of hemichannels affinity in function of their Cxs composition, poorly characterized yet.

### Contribution and safety factor

The different contribution of GJCs are a key for orchestrating the impulse propagation and cardiac function. This contribution is conjugated to the activity of active factors, specially voltage gated channels (mostly INa) and other passive factors (e.g. cell size, cytoplasmic resistance); for review: [72, 73]), that respectively provide the current source and ensure its diffusion from a stimulated *“source”* cell towards a resting *“sink”* cell. The amplitude of the current diffusing through gap junction (junctional current Ij) must be sufficient to trigger an action potential in the sink and ensure the impulse propagation [73, 74]. The amplitude of Ij varies over time in function of the voltage gating response of GJCs: as a general concept, the higher the voltage dependence of GJCs, the larger and faster the decrease of the amplitude of the source current [42]. In this sense the overall electrical properties of heteromeric, homotypic and heterotypic GJCs in a gap junction are critical, and any altered formation and properties of GJCs (becoming more or less voltage-sensitive) might represent a pro-arrhythmic risk.

Furthermore, the co-regulation between Cx43 and Na + channels (Jansen et al., 2012; Desplantez et al., 2012) has been a critical finding to better understand the triggering and sustaining of cardiac arrhythmias caused by alteration (e.g. mutations; [21, 75–78]). Indeed a reduced expression of Cx43 decreases the density of INa and the junctional content of atrial Cx45 and Cx40 [49], and most likely of ventricular Cx45. As consequence, this altered the formation and the electrical properties of GJCs [49] that exhibit pro-arrhythmic properties by slowing down the impulse propagation [18, 48, 73, 79, 80]. It appears important to elucidate how this interaction influences the Cxs co-assembling during the trafficking and the docking between hemichannels at the gap junction plaque where Na + channels and Cx43 are co-localized, and determine if a similar co-regulation exists for Cx40 and Cx45 either with Na + channel or other partner.

### Domains and motifs for Cxs assembling, GJCs formation and properties

Finally, some motifs that regulate the Cxs assembling and formation of GJCs are in same domain that regulates the functional properties of GJCs. For example the NTH domain that regulates the gating polarity of channels also contains the selectivity *signal (Cf. section B.1.3.).* How the different motifs in the same domain interact might be important for ensuring the correct formation and function of GJCs, which suggests a chronological regulation during trafficking and once inserted in the membrane. More especially the interaction with protein partners might play a role, which is still unknown and warrants further investigations.

### Future investigations: cellular approach to answers questions

This last chapter proposes future orientations of research based on our current knowledge to improve our understanding on the role of epitopes and motifs of cardiac Cxs in the formation of GJCs, correlated to their functional contribution. An important criterion to consider is the dependence on Cx level in formation of GJCs, more especially for Cx45. For this purpose cellular approaches with freshly isolated myocytes and expressing cell models (e.g. RLE cells) appear as a best option.

### Cardiac myocytes and fibroblasts

#### Atrial myocytes

In healthy adult atrial myocytes, the similar higher level of expression of atrial Cx40 and Cx43 than Cx45 should favor the Cx43/Cx40 heteromeric compatibility. On the contrary any remodeling (increased or decreased expression) should dysregulate the Cxs affinity and the hetero-oligomerization: an increase of expression of one isoform should favor its homo-oligomerization, whereas a decrease of expression might induce the homo-oligomerization of the more expressed isoform and the hetero-oligomerization of the other isoform with Cx45. So far electrical recordings revealed the hetero-oligomerization of the 3 isoforms [49], complemented with Ind40 cells that permitted to show that the Cx43/Cx40 hetero-oligomerization and contribution depend on the ratio of Cx43:Cx40 [49]. This brings the question of how Cx45, despite low level of expression, contributes to the Cxs co-assembling, if this depends on the level and ratio of the 3 isoforms or does Cx45 have a preferential affinity to Cx43 or Cx40 in function of the degree of homology between the epitopes and motifs. Importantly because pathological increase or a decrease of expression were observed, for which a striking example is AF [24, 25], further investigate this regulation in cells expressing varying levels and ratios of the 3 co-expressed Cxs in a controlled manner, similarly to our RLE models is critical (see below).

#### Ventricular myocytes

The findings and assumptions made on the regulation of formation of GJCs in ventricular myocytes (Beauchamp et al., 2004; Bao et al., 2011) is favorable to a limited Cx43/Cx45 hetero-oligomerization and a dominant population of homotypic Cx43 GJCs. Biochemically this has been quantified in the Ind45 cell line [51]), which agrees with the dependence on the level of expression of Cxs in the formation of GJCs. However electrical recordings indicated a dominant contribution of heteromeric Cx43/Cx45 GJCs [48, 50, 53, 68]. This suggests a role of Cx45 in “forcing” a low but essential co-assembling with Cx43 regulated by similar and/or different motifs and epitopes to Cx43. Interestingly the recent findings of Sorgen et al (presented at the IGJC 2015; [81]) showed that low levels of Cx45 inhibited dimerization of the COOH-Cx45 domain (residues A333-N361; [81–83]) and its homo-oligomerization in the ER in favor of a hetero-oligomerization in the TGN. Correlated to our observation in Ind45 cell line and ventricular myocytes the threshold ratio of Cx43:Cx45 can be estimated to be < 6 [12, 51]. Moreover, it might be essential to determine if this domain cross-talk with the motifs implicated in Cxs co-assembling and GJCs formation. One hypothesis could be that low levels of expression “disperse” Cx45 during the trafficking and let free the motifs for the hetero-oligomerization whereas higher levels of Cx45 might induce a “packaging” of Cx45, hide the motifs and favor the dimerization.

### Expressing cell models

The differentiation of myocytes in culture [84, 85] rend difficult the investigation of the formation of GJCs in various expression patterns of Cxs. To counteract this limit cell models as expressing system represent a positive option as they can be more easily stably transfected and maintained in long term culture than myocytes and fibroblasts. For such investigation the inducible system (Cf. Ind40 and Ind45 cell lines) that permits to mimic varying physio-pathological expression patterns is a plus.

Furthermore similarly to the current Ind40 and Ind45 cell lines we are using, the development of cell lines transfected with Cxs having altered domains (e.g. mutation, truncation) will be determinant to characterize the dependence on the level of co-expressed Cxs and if this regulates the hetero-domain interaction and Cxs co-assembling.

Until now, the formation and properties of heteromeric GJCs have been mainly performed on cell models co-expressing two Cxs. However triple Cxs co-expression is necessary to further understand the role of epitopes and motifs in Cxs co-assembling as most of cardiac tissues co-express 3 and 4 isoforms [8]. For this purpose isolated primary myocytes are determinant but as mentioned are difficult to be stably transfected. As to the transgenic animal models (i.e. Cx43^-/-^) they are essential but the cellular state of isolated myocytes results from an “all-or-none” process (embryonic development, post-birth maturation, etc.) for which the effect of the genic alteration needs to be considered but difficult to characterize. To counteract these limits the cell models appear essential and stable transfected cell lines will be a determinant tool. This requires a triple transfection in the case of using communicating deficient cell lines (e.g. HeLa) and a double transfection in a cell model that endogenously expresses one Cx (e.g. Cx43 in RLE cells). While the triple transfection is difficult to realize due to limited efficiency caused by the large amount of material to transfect, the double transfection appears more realistic. In each case the inducible system similar to our current study on the RLE model seems to be the most appropriate to control the level of co-expressed Cxs and mimic the healthy and diseased cardiac patterns of expression.

### Correlation in-vitro/in-vivo

Ultimately, the goal is to correlate cell model data with the cardiac system. This brings questions asked for several years but for which many responses have not been given yet. Among them: *are the relative formation and functional contributions of GJCs observed in* in-vitro *and ex-vivo experiments similar to what observed* in-vivo? *How can we prove their contribution?* This is a major scientific and clinical challenge that looks like an “impossible mission”. The development of computing models like the recent model of Koval [86] will be determinant for better understanding this complex machinery and open promising new therapies based on cardiac Cxs.

## ᅟ

AF: Atrial Fribrillation
AVN: Atrio-ventricular node
CH: Cytoplasmic carboxy domain
CL: Cytoplasmic loop domain
Cxs: Connexins
E1 – E2: Extracellular loops domains 1 and 2
ER: Endoplasmic reticulum
GJCs: Gap junction channels
HB: His bundle
LA: Left atria
LBB: Lower bundle branch
LV: Left ventricle
M1-M4: Transmembrane domains 1-4
NTH: Cytoplasmic amino domain
PF: Purkinje fibers
RA: Right atria
RV: Right ventricle
SAN: Sinus-atrial node
TGN: Trans-golgi network
UBB: Upper bundle branch

## Amino acid residues

Arg / R: Arginine
Asn / N: Asparagine
Asp / D: Aspartic acid
Cys / C: Cystein
Glu / Q: Glutamine
Leu / L: Leucine
Lys / K: Lysine
Trp / W: Tryptophan
